# Palliative Prognostic Index accuracy of survival prediction in an inpatient palliative care service at a Brazilian tertiary hospital

**DOI:** 10.3332/ecancer.2021.1228

**Published:** 2021-05-11

**Authors:** Mauricio Fernandes, Tiago Pugliese Branco, Maria Clara Navarro Fernandez, Carolina Paparelli, Mariana Sarkis Braz, Carolina Sassaki Kishimoto, Helena Maria de Freitas Medeiros, Karen Ebina, Luciana Regina Bertini Cabral, Simone Nagashima, Silvia Amaral de Avó Cortizo, Fabíola Borges, Mariana Ribeiro Monteiro, Ana Beatriz Kinupe Abrahao, Raphael Brandão Moreira, Alze Pereira dos Santos Tavares, Pedro Nazareth Aguiar

**Affiliations:** 1 Americas Centro de Oncologia Integrado, São Paulo 01321-001, Brazil; 2 Hospital Moriah, São Paulo 04083-002, Brazil; 3 Faculdade de Medicina do ABC, Santo André 09060-870, Brazil

**Keywords:** advanced cancer, palliative care, prognostic index, critical illness

## Abstract

**Purpose:**

The Palliative Prognostic Index (PPI) was developed to improve survival prediction for advanced cancer patients. However, there is limited data about the PPI application in a real-world scenario. This study aimed to assess the accuracy of PPI > 6 in predicting survival of cancer inpatients.

**Methods:**

A prospective observational cohort in an inpatient palliative care service at a tertiary hospital in São Paulo-SP, Brazil, between May 2011 and December 2018.

**Results:**

We included 1,376 critically ill cancer inpatients. Patients were divided into three PPI subgroups: PPI ≤ 4, PPI 4–6, and PPI ≥ 6. Their respective medium overall survival values were 44 days (95% confidence interval [CI] 35.52–52.47), 20 days (95% CI 15.40–24.59), and 8 days (95% CI 7.02–8.98), (*p* < 0.001). PPI ≥ 6 predicted survival of <3 weeks with a positive predictive value (PPV) of 72% and an negative predictive value (NPV) of 68% (sensitivity 67%, specificity 72%). PPI > 4 predicted survival of <6 weeks with a PPV of 88% and an NPV of 36% (sensitivity 74%, specificity 59%). When PPI was <4, the mortality rate over 3 weeks was 39% with a relative risk (RR) of 0.15 (95% CI 0.11–0.20; *p* < 0.001), and the 6-week mortality rate was 63% with a RR of 0.18 (95% CI 0.13–0.25; *p* < 0.001) compared to PPI ≥ 4.

**Conclusions:**

PPI was a good discriminator of survival among critically ill cancer inpatients and could assist in hospital discharge decision. PPI may help healthcare policymakers and professionals in offering high-quality palliative care to patients.

## Introduction

Physicians often overestimate the survival time of seriously ill patients. These inaccurate prognoses lead to substantial hospice underuse and later hospice enrollment in end-of-life settings [1, 2]. Hence, physicians require reliable and practical prognostic tools to improve survival prediction and optimize end-of-life care for these patients.

In recent years, the significant development and validated use of prognostic tools have improved the clinical prediction of survival [3]. These prognostic models are based on assessing laboratory variables, performance status, and other clinical variables.

The Palliative Prognostic Index (PPI) is a well-known scoring system for predicting the survival of terminally ill cancer patients, and it was initially developed in a hospice inpatient unit in Japan [4]. PPI is calculated as the sum of the scores of the following variables: Palliative Performance Score (PPS), oral intake, edema, dyspnea at rest, and delirium (Table 1). This index was validated in several settings, from palliative care units to home care settings [5–9]. Significant advantages of PPI include relying only on clinical variables without the need for laboratory measures, simple utilization, and reproducible results [10].

Therefore, we aimed to evaluate PPI accuracy for predicting the survival of inpatients at a palliative care service in a Brazilian tertiary hospital. This study assessed a substantially extensive database of patients in a real-world scenario.

## Methods

### Study design and outcomes

We conducted this prospective observational study with a cohort of terminally ill cancer in patients who were referred for a palliative care service at a tertiary hospital and assessed the accuracy of PPI in predicting the survival and discussed its potential implications in clinical practice.

The data was collected prospectively under the study.

The primary endpoint was the accuracy of PPI > 6 in predicting survival of<3 weeks and PPI > 4 predicting survival of <6 weeks. These threshold values were similar to those present in the original publication by Morita *et al* [4]. Secondary endpoints were overall survival (OS) rates at 3 weeks with PPI > 6, the most accurate PPI scores to predict 6- and 3-week survival, 3-week mortality rate with PPI > 6, and 6-week mortality rate with PPI > 4.

This study followed the ethical principles and good practices of clinical research mentioned in Helsinki Declaration and Good Clinical Practice from the ICH, and local regulations, such as the Brazilian Resolution CNS/MS 466/12 and the Document of Americas 2005. The Paulistano Hospital institutional review board approved the study protocol. The Written Informed Consent Form application was waived because this was an observational study with information collected and registered in our database and also because the study did not affect patients’ usual care.

### Patient eligibility and sampling method

The study had a convenience sampling approach with ill cancer inpatients referred for the Palliative Care Service at Paulistano Hospital, a private tertiary hospital in São Paulo-SP, Brazil, between May 2011 and December 2018.

The eligible participants could have any primary site tumor, including both hematologic and solid malignant neoplasms. Those with a potentially curable disease, i.e. candidates to definitive treatment, were considered not eligible. Eventually, we included 1,381 subjects in the study sample until December, 2018, the time to data cut-off. After excluding five patients whose PPI score was not available, 1,376 patients were included for the statistical analysis.

### Data extraction

Data regarding patients’ characteristics, primary tumor location, performance status, clinical symptoms, and PPI scores were collected within 24 hours of the first referral to palliative care service. The palliative care physicians from our hospital received specific training to conduct this assessment.

The patients were prospectively followed from the first assessment until death, decline in participation in the palliative care program, or transference to other hospitals. Patients’ survival was estimated from the first assessment to death or censored at the last contact with the care team.

Patients’ performance status was assessed with the PPS and [Eastern Cooperative Oncology Group (ECOG)/World Health Organization (WHO)] scales. According to the original publication by Morita *et al* [4] PPI was measured as the sum of the scores of the PPS, oral intake, edema, dyspnea at rest, and delirium (Table 1). The PPI total score ranges from 0 to 15, and higher scores indicate a poorer general condition and worse survival.

The oral intake was subjectively assessed as normal, moderately reduced or severely reduced by the medical evaluator. In case the patient had received total or partial parenteral nutrition, he or she has been included in the normal oral intake group. Dyspnea at rest was evaluated due patient’s symptom referral or need of supplementary oxygen. Edema was documented as any swelling of limbs with indentation after gently thumb pressure during physical exam.

Delirium was diagnosed with the criteria from the American Psychiatric Association [11]. If the delirium was provoked only by a single medication and potentially reversible with the drug withdrawal, this finding was excluded from the final score sum.

### Statistical analysis

Survival curves and 95% confidence intervals (CIs) of the median survival of the patients were calculated using the Kaplan–Meier method, and survival analyses were performed using the log-rank test. We arranged patients into three prognostic groups according to defined intervals: ≤4, 4–6, and ≥6 [4, 5].

We estimated the optimal PPI values for predicting 3- and 6-week survival based on receiver operating characteristics (ROC) curves and statistics. In this case, we included patients who either died or had a censored survival during the study.

The mortality rates at 3 and 6 weeks were evaluated according to different PPI cut-offs: PPI ≥ 6 versus PPI < 6 and PPI ≥ 4 versus PPI < 4. The statistical analysis of the odds ratio for mortality was performed using the chi-square test.

Among the patients who died, positive predictive values (PPVs) and negative predictive values (NPVs) were calculated for surviving <6 weeks with PPI > 4, and surviving <6 weeks with PPI > 6. Censored patients were not included in this analysis.

A significance level of 5% was considered for all statistical analyses. When information regarding PPI was not available, the patient was excluded from the statistical analysis for clinical outcomes. Statistical analyses were performed using IBM® SPSS Statistics version 24.

## Results

Overall, 1,381 cancer patients were included in this study; their baseline characteristics are summarized in Table 2. Most patients had a reduced ECOG performance status of 3 or 4. The most frequent primary cancers were those of the lung/chest (17.2%), colorectal (14.3%), breast (11.2%), and pancreas/hepatobiliary system (13.4%).

At the time of analysis, an actual survival date was available for 1,037 patients (75.4%) who died, whereas censored survival was registered for the remaining 338 patients (24.6%). Because six patients underwent only an initial assessment, they were excluded from the survival analysis.

**Figure 1.**
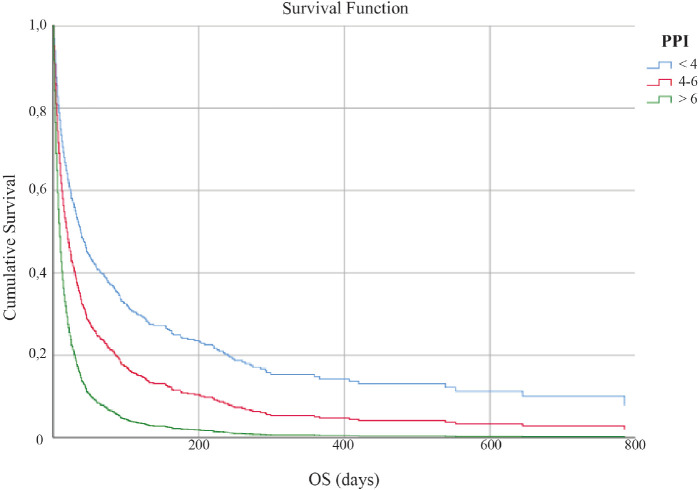
OS in days according to PPI score.

The study sample was divided into the three subgroups as follows: Group 1 included patients having PPI ≤ 4, Group 2 included those having PPI of 4–6, and Group 3 included those having PPI ≥ 6. The Kaplan–Meier survival curves for the three groups are shown in Figure 1. The median OS of Groups 1, 2, and 3 were 44 days (95% CI 35.52–52.47), 20 days (95% CI 15.40–24.59), and 8 days (95% CI 7.02–8.98), respectively, with a statistically significant difference after adjustment for primary site, age, and gender (*p* < 0.001).

### 3- and 6-week mortality rates

The primary endpoint was the accuracy of PPI > 6 in predicting survival of<3 weeks and PPI > 4 predicting survival of <6 weeks. When PPI was ≥6, the 3-week mortality rate was 86% with a RR (compared with PPI < 6) of 6.11 (95% CI 4.54–8.2; *p* < 0.001), and the 6-week mortality rate was 93% with a RR (compared with PPI < 6) of 5.44 (95% 3.72–7.96; *p* < 0.001) (Table 3).

If PPI was ≥4, the 3-week mortality rate was 81% with a RR (compared with PPI < 4) of 6.68 (95% CI 4.44–7.38; *p* < 0.001), and the 6-week mortality rate was 91% with a RR (compared with PPI < 4) of 5.61 (95% CI 3.99–7.89; *p* < 0.01). Alternatively, when PPI was <4, the mortality rate over 3 weeks was 39% with a RR (compared with PPI ≥ 4) of 0.15 (95% CI 0.11–0.20; *p* < 0.001) and that over 6 weeks was 63% with a RR (compared with PPI ≥ 4) of 0.18 (95% CI 0.13–0.25; *p* < 0.001) (Table 3).

Compared to the PPI original publication [4], in our study a PPI ≥ 6 predicted a survival of <3 weeks with a PPV of 72% and an NPV of 68% (sensitivity 67%, specificity 72%). PPI > 4 predicted a survival of <6 weeks with a PPV of 88% and an NPV of 36% (sensitivity 74%, specificity 59%) (Table 4).

### ROC curve

**Figure 2.**
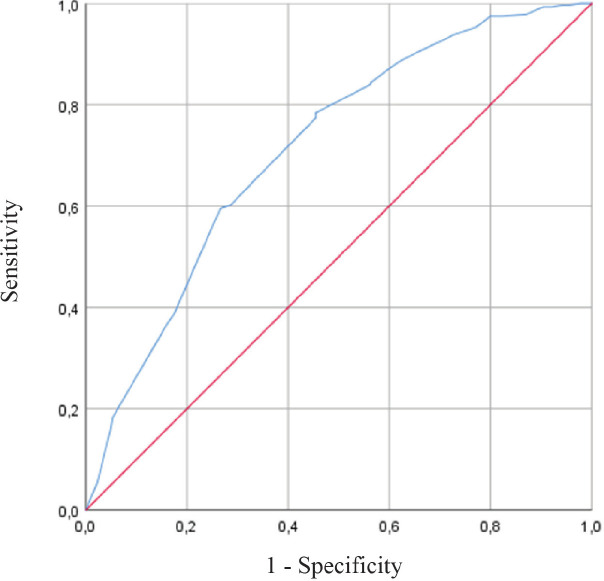
ROC Curve – PPI accuracy for survival prediction within 6 weeks.

Using ROC curves of PPI, PPI <5.5 best predicted 6-week survival with a sensitivity of 79%, a specificity of 55%, and area under the curve (AUC) of 0.714 (Figure 2). PPI ≥5.5 best predicted 3-week mortality with a sensitivity of 67%, a specificity of 73%, and AUC of 0.753.

## Discussion

### Main findings

PPI is a useful scoring system for predicting the survival of terminally ill cancer patients in several settings, such as palliative care units and home care settings [5–9]. Significant advantages of this index include relying only on clinical variables without the need for laboratory measures, simple utilization, and reproducible results [10].

A PPI single measure has good accuracy for clinical practice with terminally ill patients; however, this might be improved with serial assessments [12]. If the patient’s clinical picture further deteriorates, it may be because of an increase in the PPI value [13].

In our study, the prediction of survival of <6 weeks with PPI > 4 had high sensitivity and NPV, which were comparable to those reported in the original publication by Morita *et al* [4] However, specificity and PPV obtained in this study were slightly inferior to those reported in Morita *et al* [4] Alternatively, predicting survival of <3 weeks with PPI > 6 had inferior sensitivity and specificity values, NPVs, and PPVs compared with those reported in Morita *et al* [4]

Considering 3 and 6 weeks mortality rate, PPI equal or greater than 6 and equal or greater than 4 had almost identical results (86% and 93%. versus 81% and 91%, respectively). Conversely, the 3 weeks mortality among patients with PPI < 4 was only 39% and we consider that PPI < 4 can be considered a good prognostic factor.

Patients with PPI < 4 could likely benefit from a program and have an early hospital discharge. Moreover, clinicians should consider other factors, such as the primary site and treatment perspective, for decision-making regarding the care plan of patients with low PPI values. On the other hand, PPI ≥ 4 could be used as an indicator of a high likelihood of death and could help clinicians provide the best supportive care for patients.

We also performed a ROC curve analysis in order to find another PPI cut-off accurate and that include a larger number of patients in an early hospital discharge program validated in a future study. We found that a PPI cut-off of 5.5 would be the most accurate value for survival prediction within 3 and 6 weeks.

### Strengths and limitations

Our study’s strengths were the large sample size, prospective design, and diversity of primary cancer sites. In addition, the study database included a great number of inpatients evaluated by the palliative service in our institution and over a long-term period. This extended study duration has led the care team to a better understanding and proper use of PPI.

In contrast, our study’s limitations were the single-center study design and restriction of the study population to include inpatients under evaluation by a palliative care service from a tertiary hospital. The reality is that convenience sampling highly likely led to a biased sample, which had a predominance of seriously ill subjects and refractory symptoms. Hence, these results would be less applicable to an outpatient or hospice scenario.

However, the study context reflects the current healthcare, which is hospital-centered, expensive, and ineffective, whereas palliative care and hospice remain new concepts [14].

### Clinical relevance

This study prospectively assessed PPI’s effectiveness in a large cohort of cancer inpatients from a real-world scenario; patients enrolled in a palliative care team in a Brazilian tertiary hospital were included in this study. The routine use of PPI has been previously demonstrated to improve the clinical survival prediction of terminally ill cancer inpatients [15].

In this study, we demonstrated that PPI was a reliable and useful tool for predicting survival of critically ill cancer inpatients. Therefore, PPI will help physicians in making decisions regarding clinical practice and end-of-life care for cancer patients.

## Conclusion

PPI is a useful scoring system for predicting the survival of terminally ill cancer patients in several settings. PPI’s significant advantages include relying only on clinical variables, without the need for laboratory measures, simple utilization, and reproducible results. This study results demonstrated that PPI is a good discriminator of survival among critically ill cancer inpatients, and it could assist in the decision about hospital discharge. PPI may help healthcare policymakers and professionals in providing high-quality optimized palliative care for patients.

## Conflicts of interest and funding

All authors have no conflicts of interest to declare and the study was funded by the authors.

## Figures and Tables

**Table 1.** Description of components of PPI.

PPI	
Prognostic domains	Partial score
Palliative performance status	
10–20	4.0
30–50	2.5
≥60	0
Clinical symptoms	
Oral intake^a^	
Severely reduced	2.5
Moderately reduced	1
Normal	0
PPS	
10–20	4
30–50	2.5
≥ 60	0
Edema	
Present	1
Absent	0
Dyspnea at rest	
Present	3.5
Absent	0
Delirium^b^	
Present	4.0
Absent	0
Median survival according to PPI score	
PPI score	Median survival (days)
0–2.0	90
2.1–4.0	61
>4.0	12

Adapted from Morita *et al* [4]

a For patients receiving parenteral nutrition the score is considered zero

b Delirium is excluded if caused by a single medication

**Table 2.** Patients baseline characteristics.

Patients demographics	*N* ( %)
Number of patients	1,381
Gender	
Male	672 (48.7%)
Female	709 (51.3%)
Mean age (range)	68 years (21–100)
WHO ECOG	
0	3
1	41
2	156
3	449
4	306
NA	425
PPS	
10–20	345
30–50	861
≥60	172
NA	3
PPI	
0–2.0	108
2.1–4.0	371
>4.0	897
>6.0	525
NA	5
Survival events	1,375
Deaths	1,037 (75.4%)
Censored	338 (24.6%)
Median survival in days (95% CI)	
PPI < 4.0	44 (36–52)
4.0 ≤ PPI ≤ 6.0	20 (15–25)
PPI > 6.0	8 (7–9)
General	16 (14–18)
Primary sites	
Lung/chest	238 (17.2%)
Colorectal	198 (14.3%)
Pancreas/hepatobiliary	186 (13.4%)
Breast	155 (11.2%)
Central nervous system	107 (7.7%)
Genitourinary	107 (7.7%)
Hematological	94 (6.8%)
Upper GI	87 (6.3%)
Gynecological	78 (5.6%)
Other	131 (9.8%)

**Table 3.** Analysis of 3-weeks mortality and 6-week mortality rates according to different PPI cut-offs in the study population including patients with confirmed death (excluding censored individuals).

	3-week mortality	6-week mortality
PPI ≥ 6	Rate-86%	Rate-93%
	RR = 6.11 (95% CI 4.54–8.23)	RR = 5.44 (95% 3.72–7.96)
	*p* < 0.001	*p* < 0.001
PPI < 6	Rate-49%	Rate-71%
	RR = 0.16 (95% CI 0.12–0.22)	RR = 0.18 (95% CI 0.13–0.27)
	*p* < 0.001	*p* < 0.001
PPI ≥ 4	Rate-81%	Rate-91%
	RR = 6.68 (95% CI 4.44–7.38)	RR = 5.61 (95% CI 3.99–7.89)
	*p* < 0.001	*p* < 0.001
PPI < 4	Rate-39%	Rate-63%
	RR = 0.15 (95% CI 0.11–0.20)	RR = 0.18 (95% CI 0.13–0.25)
	*p* < 0.001	*p* < 0.001

RR refers to the comparison of the evaluated group with the complementary group (e.g. PPI < X versus PPI > X)

**Table 4.** Accuracy of predictions using the PPI – survival less than 3 weeks with PPI > 6 e survival less than 6 weeks with PPI > 4 – according to our study and Morita et al [4] original publication.

	PVV		NPV		Sensitivity		Specificity	
	Our study	Morita *et al* [4]	Our study	Morita *et al* [4]	Our study	Morita *et al* [4]	Our study	Morita *et al* [4]
PPI > 4	65%	83%	76%	71%	74%	79%	59%	77%
PPI > 6	72%	80%	68%	87%	67%	83%	72%	85%
